# G Protein‐Coupled Receptor Kinase 5 (GRK5) Modulates Nociceptin/Orphanin FQ Opioid (NOP) Receptor Desensitization in Rat Sympathetic Neurons

**DOI:** 10.1002/jnr.70110

**Published:** 2026-01-13

**Authors:** Mohamed Farrag, Marwa Soliman, Saifeldin Mahmoud, Lauren Miller, Paul B. Herold, Kristen Brandt, Victor Ruiz‐Velasco

**Affiliations:** ^1^ Department of Anesthesiology and Perioperative Medicine Penn State College of Medicine Hershey Pennsylvania USA

**Keywords:** G protein‐coupled receptor kinase, nociceptin, nociceptin/orphanin FQ opioid (NOP) receptor, N‐type voltage‐gated Ca^2+^ channel (Ca_V_2.2), stellate ganglion neurons, whole‐cell patch‐clamp technique

## Abstract

Stimulation of nociceptin/orphanin FQ peptide (NOP) opioid receptors by the endogenous ligand nociceptin (Noc) leads to voltage‐gated Ca^2+^ channel inhibition or G protein inwardly rectifying K^+^ channel activation. One mechanism of G protein‐coupled receptor (GPCR) desensitization occurs when G protein‐coupled receptor kinases (GRK) phosphorylate the agonist‐bound receptors. In the continued presence of an agonist, Gβγ recruits GRK to the plasma membrane where GPCR are then phosphorylated by GRK. The purpose of this study was to identify the GRK subtype responsible for desensitization of the Noc‐mediated Ca^2+^ current inhibition in rat stellate ganglion (SG) neurons. We observed that GRK2 and GRK5 are expressed in SG neurons. Further, silencing either GRK subtype alone or together employing siRNA did not overtly alter their Noc pharmacological profile. We assessed NOP receptor desensitization employing a protocol where the peak Ca^2+^ current inhibition was measured during intermittent application of high Noc concentrations in the continued presence of the IC_50_ Noc concentration. With this approach, we observed complete Ca^2+^ current desensitization in neurons transfected with either scrambled or GRK2 siRNA following exposure to high Noc concentrations. On the other hand, full desensitization of the Ca^2+^ currents was not observed in neurons in which GRK5 was silenced alone or with GRK2. That is, coupling of NOP receptors with Ca^2+^ channels was still observed following application of high Noc concentration. These results suggest that GRK5 plays a key role in the mechanism that mediates NOP receptor desensitization in SG neurons.

## Summary

NOP opioid receptors are expressed in sympathetic stellate ganglion (SG) neurons. We examined the role of G protein‐coupled receptor kinase (GRK) proteins in the nociceptin‐mediated NOP receptor desensitization of Ca^2+^ currents in rat SG neurons. Silencing of GRK2 or GRK5 individually or together demonstrated that Ca^2+^ current desensitization was modulated by GRK5. This is the first report to show that the membrane‐associated GRK5 isoform regulates the desensitization pathway that couples NOP opioid receptors with Ca_V_2.2 channels.

## 1 Introduction

Nociceptin/orphanin FQ peptide (NOP) opioid receptors are G protein‐coupled receptors (GPCRs) within the opioid receptor family. NOP receptors are known to regulate pain processing, cardiovascular and renal function, stress, and learning and behavior (Mogil and Pasternak 2001; Toll et al. 2016). NOP receptor activation by the endogenous ligand, nociceptin (Noc), results in G protein‐mediated inhibition of voltage‐gated Ca^2+^ (Ca_V_) channels, stimulation of G protein inwardly rectifying K^+^ (GIRK) channels, and negative coupling to adenylyl cyclase enzymes (Lambert 2008; Baiula et al. 2012). Like other opioid receptor agonists, Noc mediates its effects by coupling NOP receptors to members of the pertussis toxin (PTX)‐sensitive Gα subfamily of heterotrimeric G proteins. Our laboratory has previously reported that the NOP receptor‐mediated inhibition of Ca_V_2.2 (N‐type) channel currents in rat stellate ganglion (SG) neurons is mediated by Gα_i1_, Gβ_2_, Gβ_4_, and Gγ_7_ protein subunits (Margas et al. 2008; Mahmoud et al. 2012, 2016).

The process of G protein‐coupled receptor (GPCR) desensitization has been well‐established to result, in part, in receptor phosphorylation by G protein‐coupled receptor kinases (GRKs) (Kelly et al. 2008). In general, following receptor activation, Gβγ dimers recruit GRKs to the plasma membrane where GPCRs are phosphorylated by these kinases. Thereafter, β‐arrestin proteins interact with the phosphorylated receptors, which prevents further G protein signaling and promotes receptor internalization. Consequently, there is a lessening of the response by the cell in the continued presence of the agonist. Several studies have shown that NOP receptors internalize and desensitize in the continuous presence of selective agonists (Corbani et al. 2004; Zhang et al. 2012; Mann et al. 2019). Standifer and colleagues were the first to report NOP receptor desensitization and crosstalk with mu opioid receptors employing BE(2)‐C and SH‐SY5Y human neuroblastoma cell lines (Mandyam et al. 2002, 2003; Thakker and Standifer 2002). They found that in BE(2)‐C cells, activation of either receptor with its respective agonist led to desensitization of both receptors in a protein kinase C (PKC)‐dependent manner that also involved translocation of GRK2 and GRK3 to the cell membrane (Mandyam et al. 2002; Thakker and Standifer 2002). In a separate study, prolonged stimulation of either NOP or mu opioid receptors also resulted in desensitization of both receptor responses in BE(2)‐C and SH‐SY5Y cell lines (Mandyam et al. 2003). Another study demonstrated that the serine 363 (Ser^363^) residue of NOP receptors was critical in the GRK3‐mediated phosphorylation of the receptor in HEK293 cells (Zhang et al. 2012). More recently, it has been shown that both GRK2 and GRK3 played a key role in NOP receptor phosphorylation, internalization, and desensitization (Mann et al. 2019). Zamponi and colleagues were the first to show that NOP receptors and Ca_V_2.2 channels formed a complex that internalized following the prolonged exposure to Noc (Altier et al. 2006). In a separate study, this group found that NOP receptors formed heterodimers with mu opioid receptors and both receptor types internalized with Ca_V_2.2 in the continued presence of agonists (Evans et al. 2010).

Although the aforementioned studies addressed the question as to which GRK isoform might be involved in NOP receptor desensitization and internalization, none directly addressed the question as to which GRK isoform is responsible for the Noc‐mediated receptor desensitization regarding coupling with ion channels in neurons. Thus, in order to further our knowledge of the steps involved in NOP receptor desensitization and Ca_V_2.2 channel modulation, an experimental approach that relies on a native neuronal system that contains the signaling elements is required. The purpose of the present study was to determine the GRK protein that mediates desensitization of the Noc‐mediated Ca^2+^ current inhibition in rat SG neurons employing the whole‐cell variant of the patch‐clamp technique and siRNA transfection approaches.

## 2 Materials and Methods

### 2.1 Animals

The experiments described were approved by the Penn State College of Medicine Institutional Animal Care and Use Committee (IACUC). Male Sprague–Dawley rats (150–225 g) were initially anesthetized with CO_2_ and then rapidly decapitated with a laboratory guillotine. SG neurons were isolated as described previously (Mahmoud et al. 2012, 2016). Briefly, the SG tissue was removed and desheathed in ice‐cold Hanks' balanced salt solution. After multiple parallel slits were made perpendicular to the long axis, the SG tissue was placed in ice‐cold Opti‐MEM (Thermo Fisher Scientific) supplemented with 2 mM 2,3‐butanedione monoxime (BDM) until ready for siRNA transfection.

### 2.2 siRNA Transfection

The SG tissue was transfected with siRNA employing both electroporation and lipofection following tissue isolation and repeated once more 48 h later. For electroporation, the NEON Electroporator (Thermo Fisher Scientific) was employed. The isolated ganglia were transferred to RNase‐ and DNase‐free microcentrifuge tubes containing T solution (included in the Electroporator kit), GRK siRNA (1500–2000 nM), and 2 mM BDM. A scrambled siRNA sequence (Thermo Fisher Scientific) was employed for control experiments. The ganglia were incubated in the T solution for 10–15 min, drawn up into a 100 μL electroporator tip, and electroporated with three 20 msec 1000 V pulses. Thereafter, the ganglia were placed in a 22 mm dish containing Opti‐MEM supplemented with either scrambled (control) or GRK2 or GRK5 or GRK2&GRK5 siRNA, 2 mM BDM, and 10 μL Lipofectamine 2000 (Thermo Fisher Scientific) for 5 h in a humidified incubator (5% CO_2_/95% air) at 37°C. After the incubation period, the tissue was rinsed three times with minimal essential medium (MEM) supplemented with 10% fetal bovine serum (FBS) and 1% glutamine‐streptomycin (all from Thermo Fisher). The SG tissue was then stored in supplemented MEM. The electroporation and lipofection protocols were performed a second time 48 h after the initial transfection.

**FIGURE 1:**
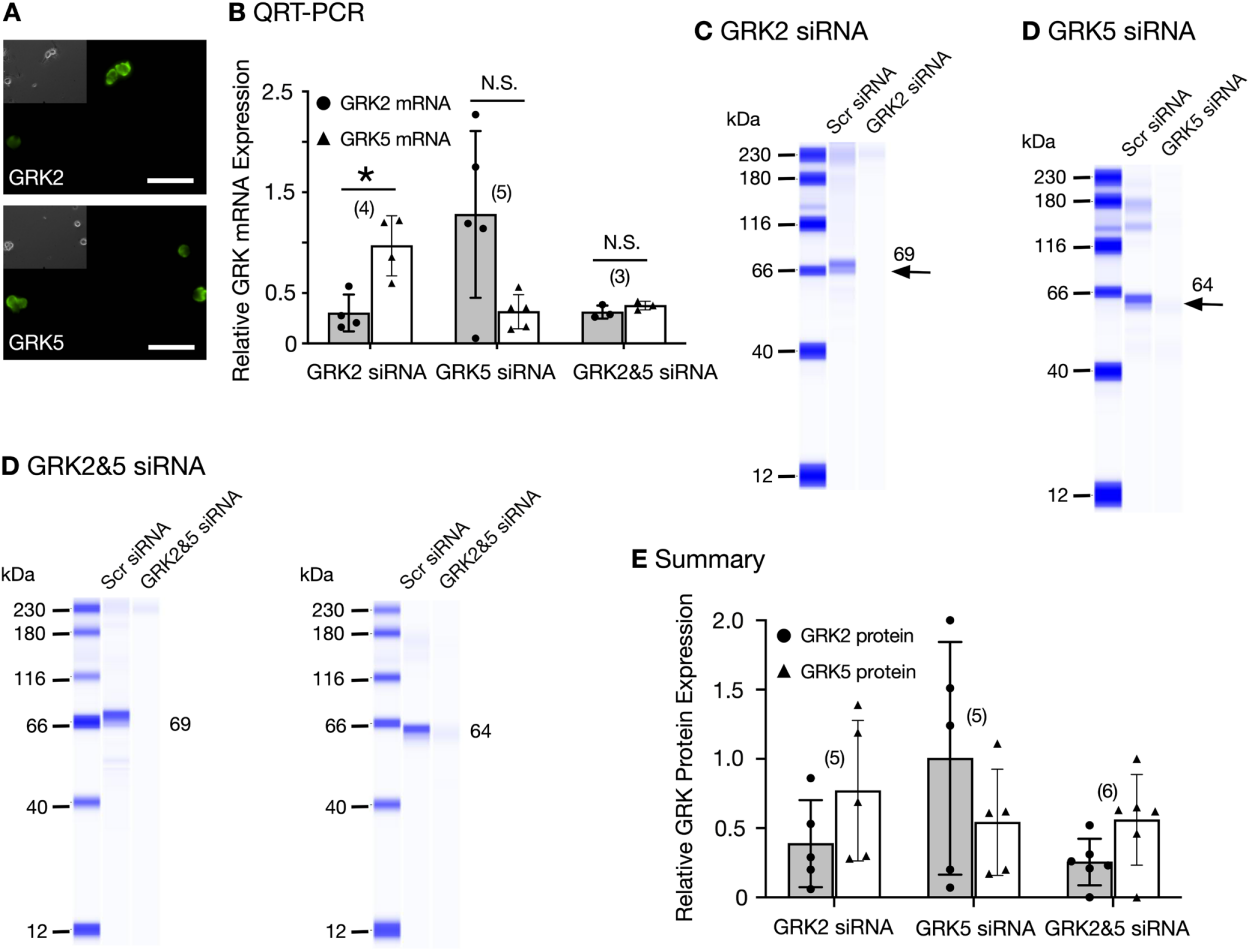
Detection of GRK2 and GRK5 expression levels 96 h post‐transfection with GRK2 or GRK5 or GRK2&5 siRNA. Immunofluorescence imaging reveals SG neurons express GRK2 and GRK5. Shown are fluorescence images of SG neurons fixed, permeabilized, and stained with primary antibodies against GRK2, followed by Alexa Fluor 488‐conjugated IgG secondary antibody. The neurons were imaged at 20× with a filter set containing an excitation filter at 480 ± 15 nm, a dichroic beam splitter of 505 nm (LP), and an emission filter at 535 ± 20 nm. The images were pseudocolored; scale bars represent 100 μm. (A) Relative expression of GRK2 (●) and GRK5 (▲) mRNA in SG tissue 96 h post‐transfection with either GRK2 siRNA or GRK5 siRNA alone or combined (B). Quantitative RT‐PCR was carried out with total RNA from scrambled siRNA‐ and GRK siRNA‐transfected SG tissue. The fold‐differences were computed by double delta *C*_t_ analysis: First, the differences between the GRK and GAPDH expression levels were calculated for each sample (∆*C*_t_), then the differences between the experimental and control groups were calculated (∆∆*C*_t_) and fold‐change determined according to the expression $2^{-∆∆C_{t}}$. * indicates *p* = 0.013 for GRK2 siRNA, while *p* values for GRK5 and GRK2&GRK5 siRNA groups were 0.138 and 0.233, respectively, and not significantly (NS) different employing the paired *t*‐test. Each point represents one animal. Representative Western blots for GRK2 (C), GRK5 (D), and GRK2&5 (E) in SG tissue 96 h post‐transfection with scrambled (Scr) or GRK2 or GRK5 or both siRNA. The 69 and 64 kDa bands represent GRK2 and GRK5, respectively. Each lane was loaded with 0.5 μg protein and represents two SG isolated from one animal. Both anti‐GRK2 and anti‐GRK5 antibodies were used at a dilution of 1:10 in the Wes system. The summary dot plot (F) shows the mean (±SD) protein levels for GRK2 and GRK5 in SG neurons transfected with GRK2, GRK5, and GRK2&5 siRNA. GRK protein levels were corrected by normalization of band area values to total protein levels for each sample. Relative expression levels between groups were computed by normalizing corrected experimental band area values to the corresponding average corrected band area of the control group. The *p*‐values for GRK2, GRK5, and GRK2&GRK5 siRNA groups were 0.197, 0.307, and 0.079, respectively, and not significantly (NS) different employing the paired *t*‐test.

The siRNA sequences employed to silence GRK2 and GRK5 were generated by employing a macro written by Stephen R. Ikeda (National Institute on Alcohol Abuse and Alcoholism) in IGOR Pro (Wavemetrics Inc.) and then chosen based on criteria described previously (Reynolds et al. 2004). The rat GRK2 target sequences were 5′‐ATGAAGAGATCGAGAAATA‐3′ and 5′‐GCAAGTGTCTCCTGCTTAA‐3′ that correspond to nucleotide positions 257–275 and 1850–1868, respectively. The rat GRK5 target sequences were 5′‐TCAGGAACATGAACTTTAA‐3′ and 5′‐GCAAACGCTTAGAGAAGAA‐3′ that correspond to nucleotide positions 1343–1361 and 641–659, respectively. The control group was transfected with scrambled siRNA (all siRNA nucleotides were obtained from Thermo Fisher Scientific). Both SG isolated from each rat were transfected with the same siRNA so that each point in Figures 1, 4 and 6 indicates one animal.

### 2.3 Quantitative Reverse Transcriptase Polymerase Chain Reaction (qRT‐PCR)

Whole rat SGs transfected with siRNA nucleotides were isolated 96 h post SG isolation and pulverized in a lysis buffer (Nucleospin RNA/Protein Kit, Macherey‐Nagel) containing the reducing agent β‐mercaptoethanol (Millipore‐Sigma), then stored at −80°C until the RNA and protein were isolated and processed. RNA samples were quantified using a Nanodrop ND‐8000 instrument (Thermo Fisher Scientific), and cDNA was made from 100 to 200 ng of RNA using the High‐Capacity cDNA Reverse Transcription Kit (Applied Biosystems). Quantitative RT‐PCR was performed using Taqman Gene Expression Master Mix, Taqman primers for GAPDH (cat. #Rn01775763_g1), GRK2 (cat. #Rn00562822_m1), and GRK5 (cat. #Rn00578086_m1) gene sequences and the QuantStudio 12 K Flex or QuantStudio 5 Real‐Time PCR System (all from Thermo Fisher Scientific).

### 2.4 Western Blotting

Protein lysates of transfected SG were prepared in lysis buffer (Macherey‐Nagel Kit) 96 h post SG isolation. The supernatant containing the protein was collected and stored at −80°C until use. Western blotting assays were performed with the “Wes” Simple Western system (Protein Simple). Once protein samples were processed, they were quantified with the Qubit 4.0 Fluorometer and the Qubit Protein Assay Kit (both from Thermo Fisher Scientific). The lyophilized master mix, which was provided in the 12–230 kDa Simple Western Separation Module (Protein Simple), was reconstituted with buffers provided in the kit. Samples were then loaded onto the Wes plate at 0.5 μg/lane.

Following size separation, the presence of GRK2 and GRK5 proteins was detected through chemiluminescence using a mouse monoclonal anti‐GRK2 primary antibody (Santa Cruz Biotechnology, cat. #sc‐13143), mouse monoclonal anti‐GRK5 primary antibody (Santa Cruz Biotechnology, cat. #sc‐518005), and a Horseradish Peroxidase‐conjugated anti‐rabbit secondary antibody provided by the manufacturer (Protein Simple, cat. #DM‐001). Both GRK antibodies were diluted to 1:10. The GRK chemiluminescence peaks were identified in the range of 60–70 kDa, and their size was determined automatically by the software through the integration of a fitted Gaussian function. Peak areas were normalized across lanes based on the measurement of total protein in each sample using an accessory kit (DM‐TP01, Protein Simple). Normalized peak areas were compared to determine the percent knockdown of GRK2 and GRK5 in siRNA‐transfected samples versus SG transfected with scrambled siRNA.

### 2.5 GRK Immunofluorescence Imaging

The dissociated SG neurons were cultured in poly‐L‐lysine‐coated 35‐mm glass‐bottom dishes (MatTek Corp.). Following overnight incubation, the cells were stained for the expression of GRK2 and GRK5 as described previously (Margas et al. 2008). The neurons were rinsed five times with 1X phosphate‐buffered saline (PBS), then fixed with 2% formaldehyde and 2% sucrose for 20 min. Thereafter, the neurons were permeabilized in 1X PBS containing 0.05% Tween 20 (EMD Biosciences) and 5% goat serum (Vector Laboratories) for 10 min at 37°C. Next, the cells were preincubated in 5% goat serum for 15 min at room temperature prior to the addition of the primary antibody. The GRK2 and GRK5 primary antibodies (described above) were diluted in 1X PBS containing 5% goat serum. Neurons were incubated with the primary antibodies for 60 min at room temperature. Following the 60‐min incubation period, the cells were preincubated in 5% goat serum for 60 min at room temperature prior to the addition of the secondary antibody. The secondary antibodies employed were Alexa Fluor 488 goat anti‐mouse (GRK2) or goat anti‐rabbit (GRK5, Thermo Fisher Scientific) at a final concentration of 3 μg/mL. The secondary antibodies were added to the cells for 45 min at 4°C. The neurons were then rinsed three times prior to imaging. The fluorescent images were acquired with a Nikon TE2000 microscope using a 20 objective, the X‐Cite 120 (EXFO Life Sciences Group) for illumination, and an Orca‐ER 1394 digital CCD camera (Hamamatsu Photonics) and iVision software (Biovision Technologies). The fluorescence images of the Alexa Fluor 488‐labeled neurons were obtained with a filter set containing an excitation filter at 480 + 15 nm, a dichroic beam splitter of 505 nm (LP), and an emission filter at 535 + 20 nm (B‐2E/C, Nikon). The fluorescence images were pseudocolored with iVision software and were postprocessed under the same conditions.

### 2.6 Electrophysiological Recordings and Data Analysis

Ninety‐six hours after siRNA transfection (day before recording), the SG tissue was enzymatically dissociated in Opti‐MEM containing 0.6 mg/mL collagenase (Roche Applied Science), 0.4 mg/mL trypsin (Worthington Biochemical), and 0.1 mg/mL DNase (Millipore‐Sigma) in a shaking water bath at 35°C for 60 min. The neurons were next dispersed by vigorous manual shaking, centrifuged twice for 6 min at 63 *g*, and resuspended in supplemented MEM (described above). Finally, the neurons were plated onto 35 mm poly‐L‐lysine‐coated dishes and stored in a humidified incubator at 37°C overnight.

Following overnight incubation, Ca^2+^ currents were acquired with the whole‐cell patch‐clamp technique. The patch pipet electrodes (No. 8250; King Precision Glass) were pulled on a P‐97 micropipette puller (Sutter Instrument Co.), coated with Sylgard (Dow Corning), and fire polished on a microforge. The Ca^2+^ currents were acquired with an Axopatch 200B amplifier (Molecular Devices), analog filtered at 1–2 kHz (−3 dB, four‐pole, low‐pass Bessel filter), and digitized with custom‐designed F6 software (Stephen R. Ikeda, National Institute on Alcohol Abuse and Alcoholism) equipped with an 18‐bit AD converter board (HEKA Instruments Inc.). The cell membrane capacitance and pipette series resistance were electronically compensated (80%–85%).

**FIGURE 2:**
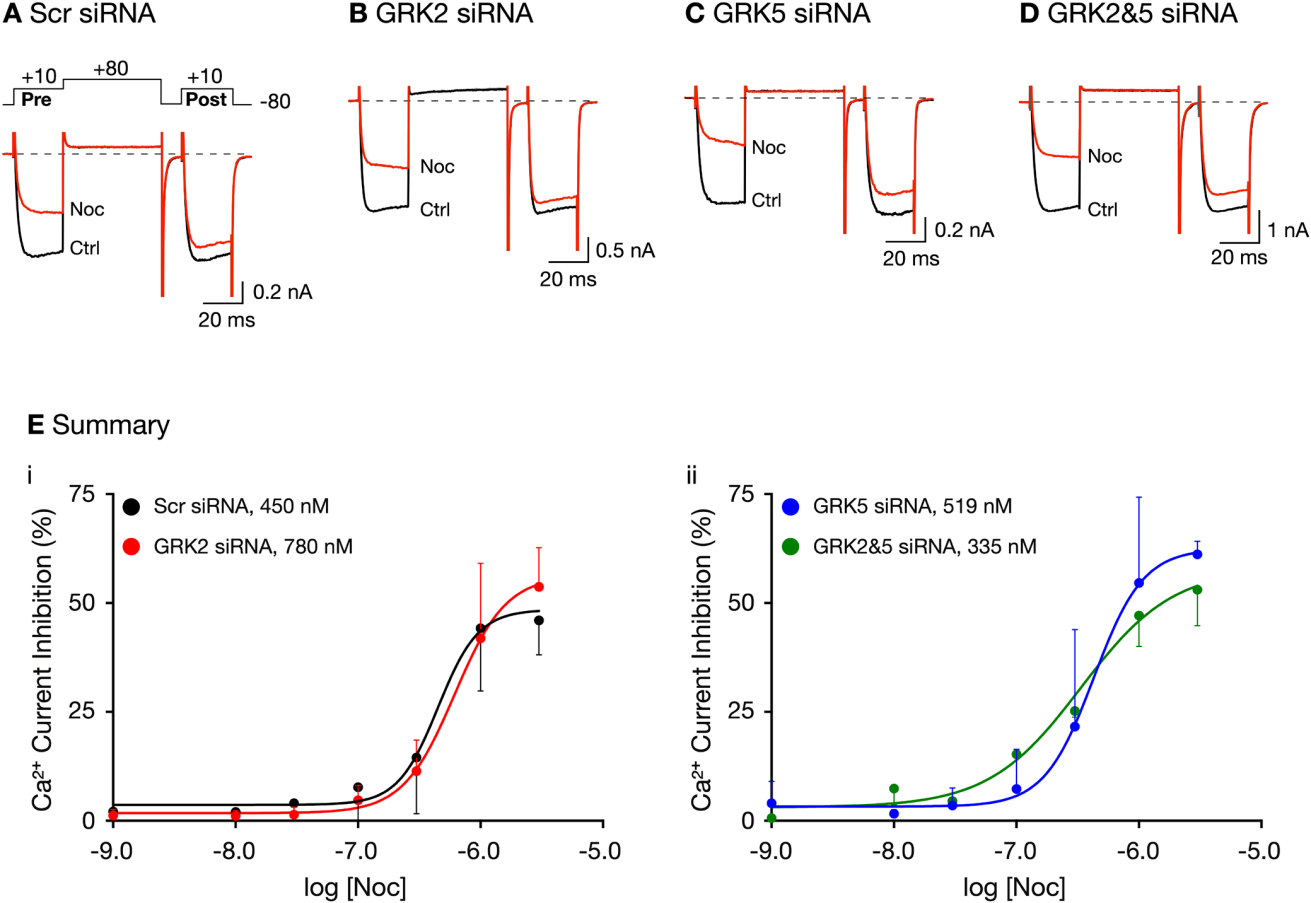
Noc concentration–response relationships in SG neurons 96 h post‐transfection with GRK2 or GRK5 or GRK2&5 siRNA. Representative prepulse and postpulse Ca^2+^ current amplitude acquired from the application of Noc in SG neurons transfected with Scr (A), GRK2 (B), GRK5 (C), and GRK2&5 siRNA. Currents were evoked every 10 s with the “triple‐pulse” voltage protocol (A, top). Black traces represent control currents (before Noc exposure), and red traces represent inhibition of currents during 1.0 μM Noc exposure. Noc concentration–response curves in SG neurons transfected with scrambled, Scr, (●) and GRK2 (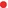) (Ei), and GRK5 (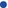) and GRK2&GRK5 siRNA (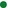) (Eii). Each data point represents the mean (±SD, *n* = 3–20) Ca^2+^ current inhibition. A total of nine animals were employed. The smooth curves were obtained by fitting the points to the Hill equation, and IC_50_ values for each group are indicated in the inset.

**FIGURE 3:**
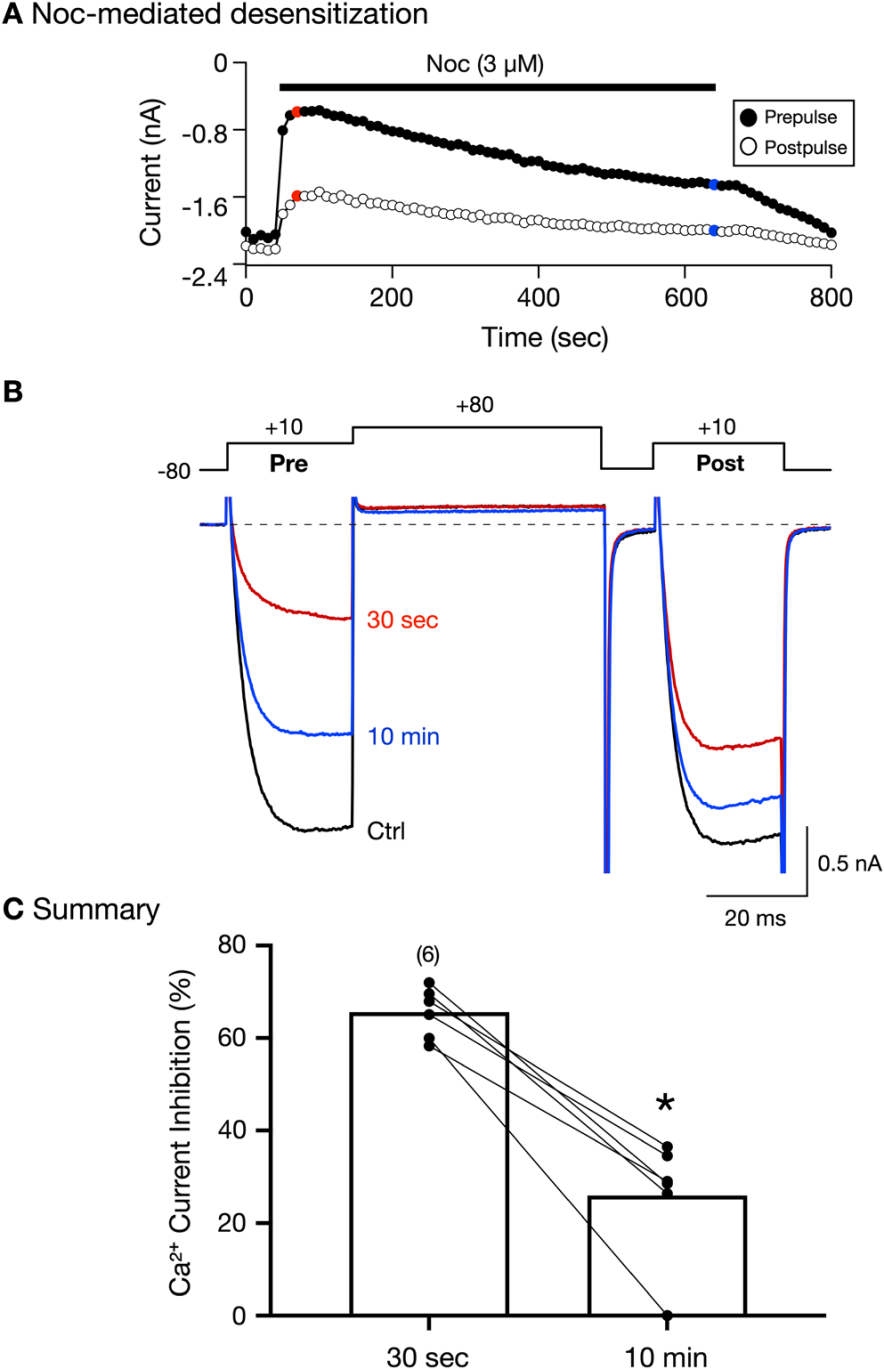
Noc‐mediated desensitization of Ca^2+^ current inhibition mediated by a high concentration (3 μM) of Noc and recovery from desensitization in SG neurons. Time course of peak Ca^2+^ current amplitude for prepulse (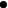) and postpulse (◯) acquired before and during the application of 3 μM Noc in an acutely isolated SG neuron. Currents were evoked every 10 s with the “triple‐pulse” voltage protocol (Figure 3B, top). The current traces represent those obtained before (Ctrl, black trace) and 30 s (red trace) or 10 min (blue trace) during Noc application. C, summary dot plot showing the mean (±SD) prepulse Ca^2+^ current inhibition mediated by Noc (3 μM) following a 10 min exposure. The numbers in parentheses indicate the number of neurons tested and obtained from two animals. * indicates *p* = 0.0004 employing the paired *t*‐test.

**FIGURE 4:**
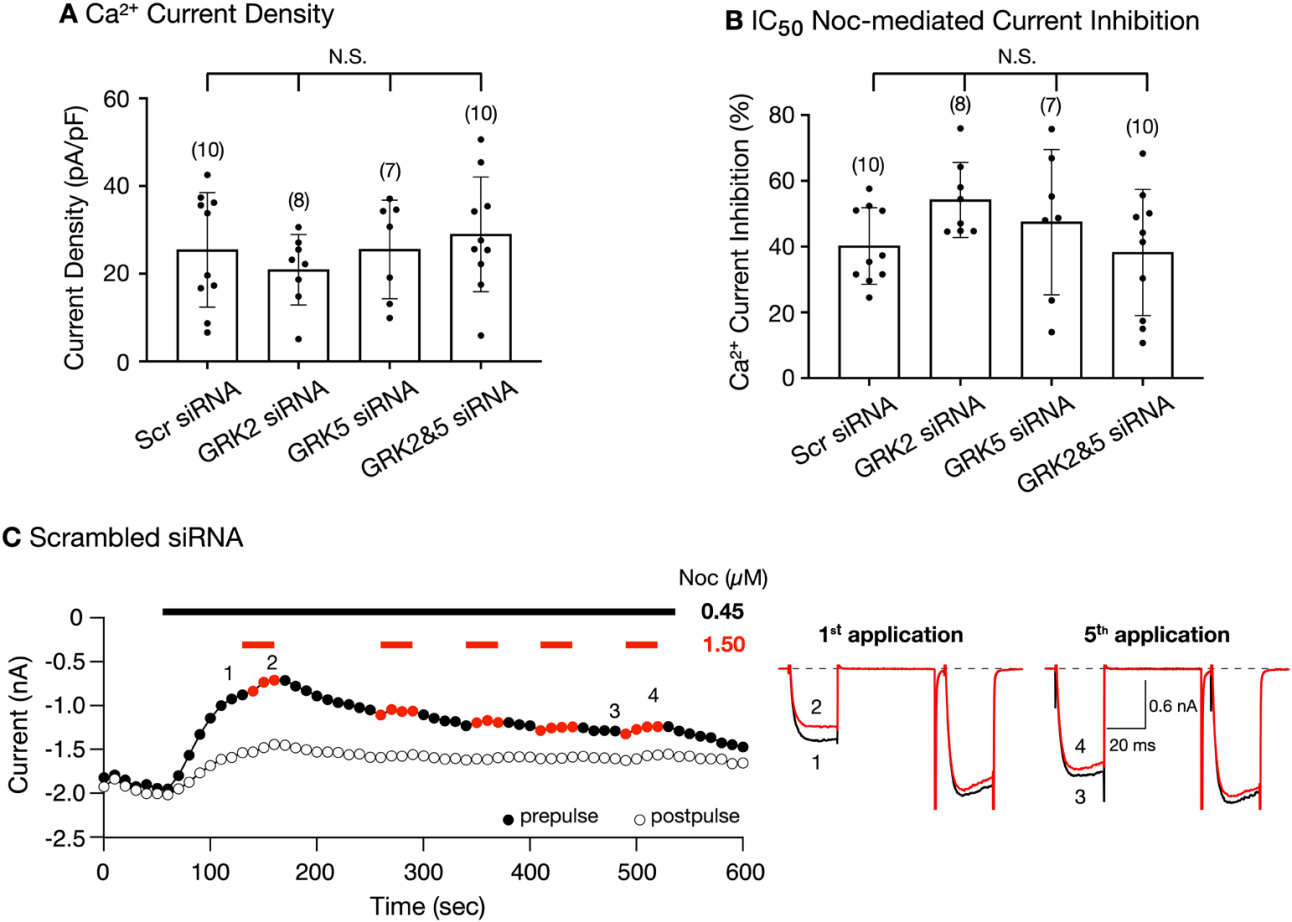
Ca^2+^ current density and desensitization of Ca^2+^ current inhibition mediated by a high concentration of Noc in scrambled siRNA‐transfected SG neurons. Measurement of the mean (±SD) Ca^2+^ current density (A) and IC_50_ (0.45 μM) Noc‐mediated current inhibition at the depolarizing potential to +10 mV (B). The current density was calculated from the peak Ca^2+^ current amplitude at the test pulse of +10 mV and normalized to the membrane capacitance. The numbers in parentheses indicate the number of neurons tested. Neither the mean current density nor the current inhibition for all GRK siRNA‐transfected groups was significantly (NS) different (*p* > 0.05) from SG neurons transfected with scrambled siRNA (one‐way ANOVA, Dunnett's multiple comparison test). Time course of peak Ca^2+^ current amplitude for prepulse (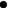) and postpulse (◯) acquired from the continuous application of a Noc IC_50_ (black line) or high and IC_50_ concentrations (~3× IC_50_, red lines) in SG neurons transfected with scrambled siRNA (C). Currents were evoked every 10 s with the “triple‐pulse” voltage protocol (Figure 3B, top). The numbered Ca^2+^ current traces are shown to the right, where 1 and 3 (black) represent currents obtained in the presence of an IC_50_ Noc concentration (0.45 μM), and 2 and 4 (red) represent currents obtained in the presence of high (1.5 μM) and IC_50_ Noc concentrations.

NOP receptor inhibition of Ca^2+^ currents was examined employing the triple‐pulse protocol (Ikeda 1991; Lu and Ikeda 2016). For this voltage paradigm, shown in Figure 2A (top), a 25 msec test pulse (prepulse) to +10 mV is followed by a depolarizing 50 msec conditioning test pulse to +80 mV, then a return to −80 mV, followed by a second 25 msec test pulse (postpulse) to +10 mV and finally returning to −80 mV. The peak Ca^2+^ current amplitude for prepulse and postpulse currents was measured isochronally 10 msec following the initiation of each pulse. The prepulse serves as a baseline measurement of initial activation of Ca_V_2.2 channels. The conditioning test pulse relieves inhibition via dislodging inhibitory G protein subunits from Ca_V_2.2 channels. The postpulse measures the Ca^2+^ current after the dislodging step to determine whether there is a change in the amplitude or other properties relative to the first pulse. The ratio of the postpulse to the prepulse current amplitude, known as the facilitation ratio (FR, Figure 6B), is a criterion typically used to examine the Gβγ subunit‐mediated voltage‐dependent Ca^2+^ channel inhibition (Lu and Ikeda 2016). Employing this procedure allows us to measure basic Ca_V_2.2 channel function during experimental manipulations, ensuring that any interventions, such as siRNA knockdown, do not inadvertently affect fundamental Ca_V_2.2 channel properties (Exp. 2.1). The Ca^2+^ current density (Figure 4A) was determined by obtaining the peak Ca^2+^ current amplitude acquired at the test pulse of +10 mV and then normalizing to membrane capacitance. Furthermore, the membrane capacitance was calculated from the numerical integration of a transient recorded during a depolarizing pulse from −80 mV to −70 mV before electronic compensation. For the desensitization measurements, the Noc‐mediated Ca^2+^ current inhibition (plotted in Figures 2, 3, 4 and 6, denoted Ca^2+^ current inhibition hereafter) was calculated as follows: (peak prepulse current [before Noc] – peak prepulse current [after Noc])/(peak prepulse current [before Noc]) × 100.

The Noc concentration–response relationships were determined by the sequential application of the peptide at increasing concentrations. Each neuron was exposed to two different concentrations. The results for each group were then pooled, and the curves were fit to the Hill equation: *I* = *I*_MAX_/{1 + (IC_50_/[Noc])^nH^}, where *I* is the percent inhibition, *I*_MAX_ is the maximum Ca^2+^ current inhibition, IC_50_ is the half‐maximum Noc concentration, [Noc] is the Noc concentration of the test solution, and nH is the Hill coefficient. These parameters were obtained using the Prism 10.0 software package (GraphPad Software Inc.). Thereafter, the fits to the concentration–response relationships were compared employing the extra‐sum‐of‐squares *F* test.

The external bath solution consisted of (in mM): 140 methanesulfonic acid, 145 tetraethylammonium hydroxide (TEA‐OH), 10 HEPES, 15 glucose, 10 CaCl_2_, and 0.0003 tetrodotoxin. TEA‐OH was used to adjust the pH to 7.4. The pipette solution contained (in mM): 20 TEA‐OH, 80 N‐methyl‐D‐glucamine, 20 CsCl, 40 CsOH, 11 EGTA, 10 HEPES, 1 CaCl_2_, 4 MgATP, 0.3 Na_2_GTP, and 14 Tris‐creatine phosphate. Methanesulfonic acid was used to adjust the pH to 7.2. Stock solutions of Noc (Tocris Cookson) were prepared in water and diluted in the bath solution to their final concentration prior to use. A gravity‐fed perfusion device was positioned ~100 mm from the neuron for agonist application.

### 2.7 Statistical Analysis

Data and statistical analyses were carried out with Igor Pro 6.0 (Wavemetrics Inc.) and Prism 7.0 software packages. A *p* < 0.05 was considered statistically significant. Graphs and current traces were generated with Graphic 3.0 software (Picta Inc.).

## 3 Results

### 3.1 Silencing GRK2 and GRK5 in SG Tissue

The purpose of the present study was to identify the GRK proteins involved in the NOP receptor desensitization, assessed via Ca^2+^ current modulation. Initial immunofluorescence assays indicated that SG neurons expressed GRK2 and GRK5 proteins (Figure 1A). Thus, in the first set of experiments, SG tissue was transfected with GRK2 and GRK5 siRNA. Our initial experiments indicated that a period of 96 h was necessary to obtain significant silencing of both proteins. Figure 1A is a scatter plot representing the GRK mRNA expression in SG tissue transfected with either GRK2 or GRK5 or GRK2&GRK5 siRNA. It can be observed that SG tissue transfected with GRK2 siRNA had a mean of approximately 30% expression of GRK2 mRNA (●) compared to SG tissue transfected with scrambled siRNA. Also, in this group, GRK5 mRNA (▲) expression levels were 97% compared to scrambled siRNA‐transfected SG tissue (*n* = 4 rats). The plot also indicates that SG tissue transfected with GRK5 siRNA showed that GRK5 mRNA (▲) expression levels were 25% compared to scrambled siRNA‐transfected tissue (*n* = 5 rats). Also note that in the same group of animals, the mean GRK2 mRNA (●) levels remained relatively unchanged in SG tissue from two animals, while the levels increased in the tissue of one rat and decreased in another animal when compared to scrambled siRNA‐transfected SG tissue (Figure 1A). The decrease may have resulted from off‐target actions by the siRNA in this specific tissue (discussed below). Finally, the SG tissue transfected with both GRK2 and GRK5 siRNA showed that mRNA expression levels for GRK2 (●) and GRK5 (▲) decreased to 31 and 38%, respectively, when compared to tissue transfected with scrambled siRNA (*n* = 3 rats).

GRK2 and GRK5 protein levels were also determined in the transfected SG tissues. Figure 1B shows a Western blot testing for GRK2. SG tissue transfected with scrambled siRNA detected GRK2 expression (~69 kDa), while it was mostly absent in GRK2 siRNA‐transfected SG tissue. Similarly, Figure 1C shows a Western blot of SG tissue transfected with either scrambled or GRK5 siRNA. The lane loaded with the GRK5 siRNA sample shows little GRK5 (~64 kDa) amount compared to tissue transfected with scrambled siRNA. SG tissue transfected with both GRK2 and GRK5 is shown in Figure 1D. The blot on the left in Figure 1D indicates that GRK2 was decreased when compared to the tissue transfected with scrambled siRNA, while the blot on the right shows similarly that GRK5 expression is less in SG tissue transfected with GRK2 and GRK5 siRNA when compared to that transfected with scrambled siRNA. The summary plot (Figure 1E) depicts GRK2 and GRK5 expression levels in SG neurons transfected with scrambled siRNA or with siRNA targeting either GRK isoform alone or in combination. In some cases, it can be observed that transfection with either GRK2 or GRK5 siRNA resulted in incomplete knockdown of the intended GRK isoform (discussed below).

### 3.2 Noc Pharmacological Profiles

Prior to investigating the effects of GRK silencing on NOP receptor desensitization, we first determined whether silencing either GRK subtype alone or together would alter NOP receptor coupling to Ca_V_2.2. This step ensures that the siRNAs do not unintentionally alter NOP receptor coupling to Ca_V_2.2, which would otherwise skew the interpretation of the desensitization experiments. In SG neurons, Ca_V_2.2 channels carry most of the Ca^2+^ (Gilbert et al. 1998; Kukwa et al. 1998; Fuller et al. 2004). Ca^2+^ currents were evoked every 10 s with the triple‐pulse protocol (shown on top of Figure 2A and described in the Methods: Section 2), and Ca^2+^ current inhibition was measured following exposure to Noc. The Ca^2+^ current traces shown in Figure 2A were obtained from an isolated SG neuron transfected with scrambled siRNA. Prior to agonist application, the Ca^2+^ current amplitude for both prepulse and postpulse was 0.93 and 0.95 nA, respectively (black trace). Thereafter, the neuron was exposed to 1 μM Noc (red trace), and the prepulse Ca^2+^ current decreased to 0.51 nA (~45% inhibition), and its rising phase exhibited kinetic slowing. This latter effect is thought to result from a voltage‐dependent relief of block during the test pulse (Ikeda and Dunlap 1999). Additionally, it is evident that during exposure to Noc, the amplitude of the postpulse current (0.85 nA) was greater than that measured during the prepulse (0.51 nA), a phenomenon referred to as facilitation of Ca^2+^ currents. That is, prior to Noc exposure, the conditioning pulse to +80 mV did not overtly alter the postpulse amplitude (black trace). On the other hand, during Noc application, the postpulse amplitude (red trace) was greater than that observed for the prepulse, and there is “relief” of the Noc‐mediated block following the conditioning pulse. Large FR values (typically > 1.3) are indicative of voltage‐dependent modulation that is observed with Ca_V_2.2 channels.

Figure 2B–D shows Ca^2+^ current traces from dissociated SG neurons transfected with GRK2, GRK5, and GRK2&5 siRNA, respectively, and exposed to 1 μM Noc. Similar to the neuron transfected with scrambled siRNA, exposure of the three SG neurons to 1 μM Noc resulted in a 40% or greater inhibition of the Ca^2+^ currents. It can also be observed that, in all three cells, there was a voltage‐dependent inhibition and facilitation of the Ca^2+^ currents. Figure 2Ei,Eii depict the Noc concentration–response curves for all SGs transfected with siRNA. The measured data points were fit to the Hill equation and the IC_50_ values for the four groups of SG neurons are provided in the figure inset. The plots show that siRNA transfection did not lead to significantly overt changes in coupling between NOP receptors and Ca_V_2.2 channels.

### 3.3 Ca^2+^ Current Desensitization

The time course in Figure 3A depicts the prepulse (●) current amplitude and postpulse (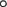) current amplitude before and during a 10 min exposure (solid bar) to Noc (3 μM) in a control SG neuron. The superimposed traces shown in Figure 3B correspond to the Ca^2+^ currents acquired before Noc exposure (Ctrl, black trace), and 30 s (red trace) and 10 min (blue trace) following Noc application. It can be observed that within 30 s, the maximum Ca^2+^ current inhibition (72%) was obtained, while at the end of the 10 min Noc exposure, the inhibition of the currents (29%) is less than that observed acutely at 30 s. Further, the voltage‐dependent inhibition is more pronounced at 30 s when compared to that measured at 10 min. The summary scatter plot in Figure 3C is a comparison of the Noc‐mediated Ca^2+^ current inhibition for the 30 s and 10 min time points. The inhibition of Ca^2+^ currents was significantly (*p* = 0.004) greater for the former than for the latter, indicating desensitization.

### 3.4 Ca^2+^ Current Desensitization in the Presence of an Noc IC_50_ Concentration in Scrambled siRNA‐Transfected SG Neurons

The next set of experiments focused on identifying which GRK subtype is responsible for the Noc‐mediated desensitization of Ca^2+^ currents by employing a so‐called “low dose protocol” designed by Williams and colleagues (Alvarez et al. 2002). In that study, the authors employed three separate protocols to measure mu opioid receptor desensitization: two “high dose” and one “low dose.” We did not employ the “high dose” protocols because the recovery period we observed following a short exposure (~30 s or less) to a high Noc concentration (> 1 μM) typically takes more than 3 min (Ruiz‐Velasco et al. 2005; Mahmoud et al. 2010). On the other hand, the advantage of employing the “low dose protocol” is that rapid Noc washout is not necessary. Thus, with the “low dose protocol,” the SG neurons were continuously exposed to a submaximal (i.e., IC_50_) Noc concentration. Additionally, a higher concentration (i.e., 3X IC_50_) was then applied intermittently (to avoid desensitization) at various intervals for a total of five applications. We first determined whether silencing either GRK isoform alone or together would result in an alteration of the Ca^2+^ current density. Figure 4A is a summary plot of the Ca^2+^ current densities in SG neurons transfected with scrambled, GRK2, GRK5, and GRK2 and GRK5 siRNA. The *n* values for each group are indicated on the graph in parentheses. The mean values between all groups were not significantly different (one‐way ANOVA and Dunnett multiple comparisons test, *p* = 0.56). Similarly, we compared the Noc IC_50_‐mediated current inhibition for the four SG groups. The summary plot in Figure 4B shows the mean (±SD) Ca^2+^ current inhibition following exposure to Noc IC_50_. The *n* values for each group are indicated in parentheses. A comparison of mean values showed that there was no significant difference among the four groups (one‐way ANOVA and Dunnett multiple comparisons test, *p* = 0.18).

We then measured the inhibition of the prepulse Ca^2+^ current during exposure to the higher Noc concentration. Given the varying IC_50_ values determined for each group, the high Noc concentration employed in this set of experiments was approximately three times the IC_50_ of the siRNA‐transfected SG neurons (Figure 2Ei,Eii). The application of the high concentration was limited to no more than 40 s to avoid desensitization. A decrease in prepulse current inhibition produced by the high Noc concentration on the fifth application was taken as a measure of desensitization (Alvarez et al. 2002). The time course of Ca^2+^ current block in Figure 4C illustrates this approach in an SG neuron transfected with scrambled siRNA. Based on the pharmacological profile results shown in Figure 2E, the neuron was continuously exposed to 0.45 μM Noc. Prior to Noc application, the Ca^2+^ current amplitudes for the prepulse (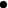) and postpulse (◯) were 1.95 and 2.0 nA, respectively. After exposure to 0.45 μM Noc (black dash), the prepulse current was blocked by 52% (0.9 nA, prepulse trace 1, ●). Following the first application of the high Noc concentration (1.50 μM, red dash), the current was blocked by 23% (prepulse trace 2, 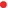). Following the removal of the high Noc concentration, it can be observed that the prepulse Ca^2+^ current amplitude began to recover with a value of 1.0 nA prior to the second exposure to the high Noc concentration. The high Noc was applied intermittently four additional times. Prior to the fifth exposure to the high concentration, the prepulse Ca^2+^ current amplitude was approximately 1.32 nA (prepulse trace 3, ●). After the fifth application of the high concentration, the Ca^2+^ current inhibition was 6% (trace 4, 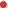), suggesting that there was less coupling between NOP receptors and Ca_V_2.2 channels. Note that the prepulse current amplitude kept increasing throughout the exposure to Noc, indicative of a desensitized response. On the other hand, the postpulse current amplitude remained relatively constant before and during the application of high Noc. The summary plot in Figure 6A is a comparison of the high concentration Noc‐mediated prepulse Ca^2+^ current inhibition or enhancement between the first and fifth application for the four siRNA‐transfected SG groups. A repeated measures two‐way ANOVA analysis showed that in the scrambled siRNA‐transfected neurons, the Noc‐mediated inhibition of the prepulse current on the fifth application (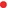) was significantly (*p* = 0.006) lower than that observed during the first Noc application (●).

### 3.5 Ca^2+^ Current Desensitization in the Presence of an Noc IC_50_ Concentration in GRK2 siRNA‐Transfected SG Neurons

**FIGURE 5:**
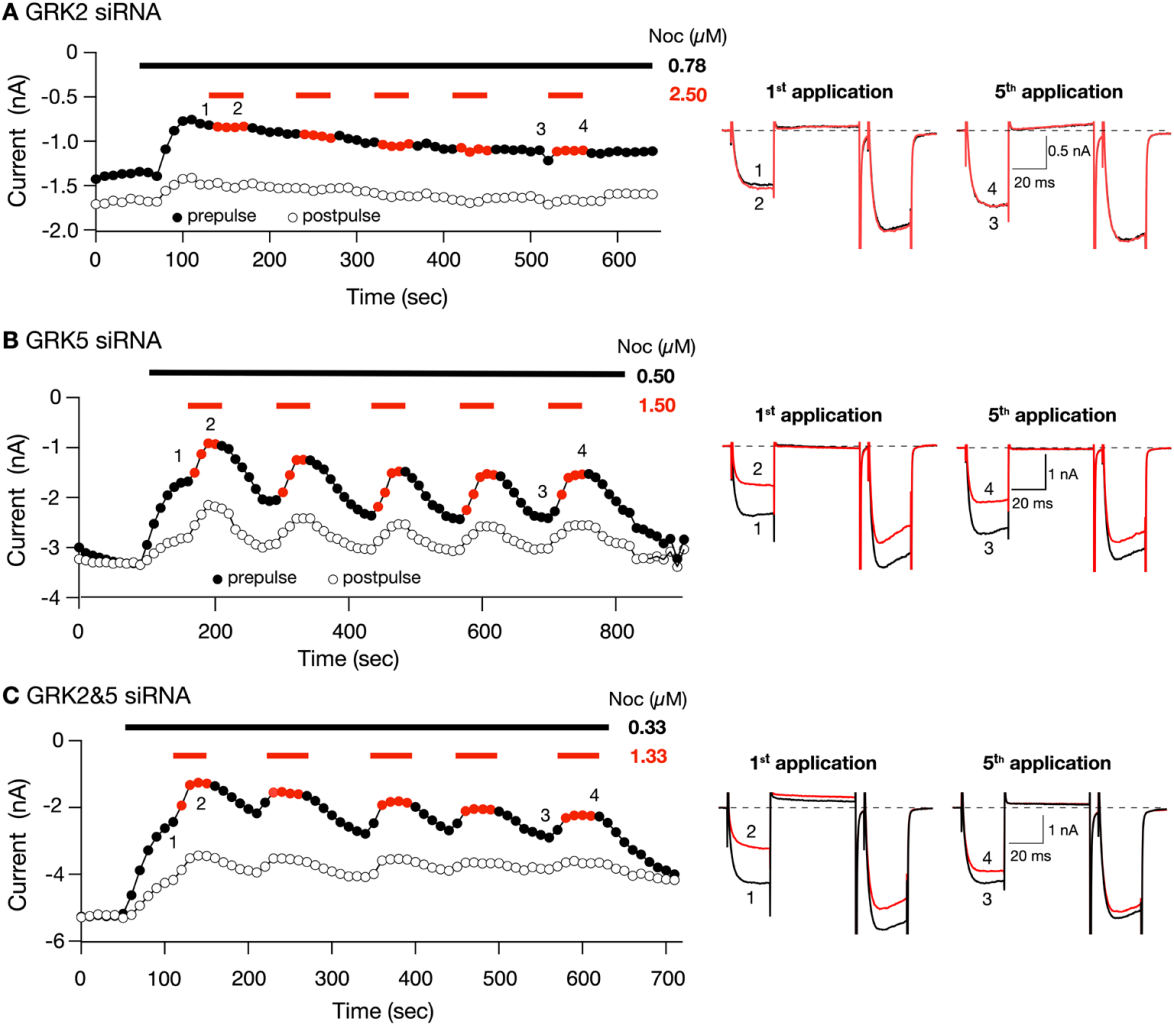
Effect of GRK2 or GRK5 or GRK2&5 silencing on the desensitization of Ca^2+^ current inhibition mediated by a high concentration of Noc in SG neurons. Time courses of peak Ca^2+^ current amplitude for prepulse (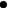) and postpulse (◯) acquired from the continuous application of a Noc IC_50_ (black line) or IC_50_ and high concentrations (~3× IC_50_, red lines) in SG neurons transfected with GRK2 siRNA (A), GRK5 siRNA (B), and GRK2&5 (C). Currents were evoked every 10 s with the “triple‐pulse” voltage protocol (Figure 3B, top). The numbered Ca^2+^ current traces for each group are shown to the right, where 1 and 3 (black) represent currents obtained in the presence of an IC_50_ Noc concentration, and 2 and 4 (red) represent currents obtained in the presence of both IC_50_ and high Noc.

**FIGURE 6:**
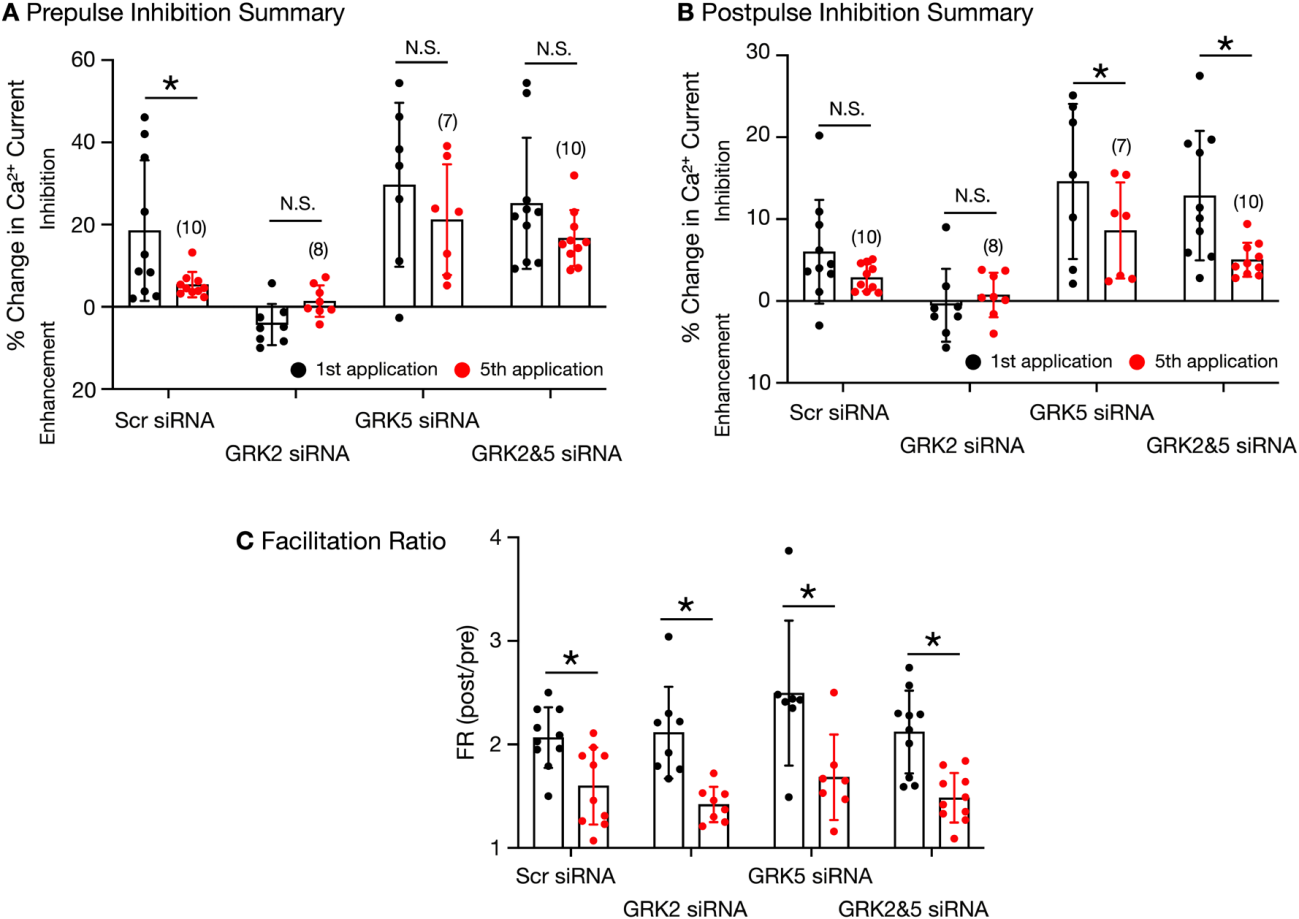
Summary of the prepulse and postpulse Ca^2+^ current inhibition mediated by a high Noc concentration in SG neurons and facilitation ratios. Summary dot plot of mean (±SD) % change in prepulse Ca^2+^ current (A) and postpulse Ca^2+^ current (B) following the first (●) and fifth (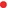) exposure to a high concentration of Noc in SG neurons transfected with scrambled, GRK2, GRK5, and GRK2&5 siRNA. The numbers in parentheses indicate the number of neurons tested and were obtained from seven animals. The *p*‐values obtained were 0.14, 0.59, 0.02, and 0.001 for scrambled, GRK2, GRK5, and GRK2&GRK5 siRNA groups, respectively. Mean (±SD) facilitation ratio (FR) obtained from SG neurons transfected with siRNA (C). FR was calculated as the ratio of the postpulse to prepulse Ca^2+^ current amplitude acquired during the first (●) and fifth (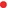) Noc exposure. The groups in A through C were compared with the repeated measures two‐way ANOVA followed by Bonferroni's multiple comparison test. NS and * indicate not significantly different and *p* < 0.05, respectively.

Figure 5A is a time course of Noc Ca^2+^ current block in an SG neuron transfected with GRK2 siRNA. After the Noc IC_50_ (0.78 μM) was applied (black dash), the prepulse Ca^2+^ current decreased from 1.36 to 0.75 nA, a 45% current block. Following exposure to the first high concentration (2.5 μM, red dash), we observed an 10% enhancement of the prepulse Ca^2+^ current from 0.75 nA (trace 1, ●) to 0.83 nA (trace 2, 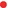). Following the fifth exposure to high Noc, the prepulse current decreased 1.2% from 1.12 nA (trace 3, ●) to 1.10 nA (trace 4, 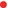). Note that similar to the time course described for the scrambled siRNA group above (Figure 4C), the prepulse Ca^2+^ current amplitude kept increasing in the presence of the IC_50_ Noc, suggesting desensitization had occurred. It can also be observed that the postpulse current amplitude remained relatively constant before and during high Noc exposure. Figure 6A shows that in the GRK2 siRNA‐transfected neurons, the first exposure (●) to high Noc concentration led to an average enhancement of the prepulse Ca^2+^ currents of 4.3%, while the fifth application (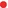) led to minimal inhibition of 1.2%. The mean (±SD) Noc‐mediated inhibition values observed for first and fifth applications were not significantly different (repeated measures two‐way ANOVA, *p* = 0.26).

### 3.6 Ca^2+^ Current Desensitization in the Presence of an Noc IC_50_ Concentration in GRK5 siRNA‐Transfected SG Neurons

Figure 5B shows the time course in an SG neuron transfected with GRK5 siRNA. The IC_50_ application (black dash) resulted in a block of prepulse Ca^2+^ currents by 49% (from 3.3 to 1.7 nA). During exposure to the high concentration (1.5 μM, red dash), the prepulse current (black trace 1, ●) was blocked by 46% (red trace 2, 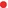). After removal of the high Noc concentration, the prepulse current amplitude began to recover. The time course also shows that, unlike the scrambled and GRK2 siRNA‐transfected neurons, exposure to high Noc for the second to fifth applications resulted in coupling of NOP receptors to Ca_V_2.2 channels that did not exhibit desensitization. Thus, a comparison of the mean (±SD) Noc‐mediated Ca^2+^ current inhibition observed for the first and fifth applications indicated no significant difference (repeated measures two‐way ANOVA, *p* = 0.17). It can be seen that the postpulse current amplitude also decreased during exposure to high Noc concentrations. These results indicate that the coupling between NOP receptors and Ca^2+^ channels remained almost consistent between the first and fifth Noc exposure.

### 3.7 Ca^2+^ Current Desensitization in the Presence of an Noc IC_50_ Concentration in GRK2 and GRK5 siRNA‐Transfected SG Neurons

The time course shown in Figure 5C is that of an SG neuron transfected with GRK2 and GRK5 siRNA. Prior to IC_50_ Noc (0.33 μM, black dash) application, the prepulse current amplitude was 5.2 nA and blocked by 50% (2.6 nA) following Noc exposure. The time course also shows that the initial application of high Noc concentration (1.33 μM, red dash) blocked the prepulse Ca^2+^ currents by 50% (from 2.6 to 1.3 nA, see traces 1, ● and 2, 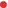). Following the fifth application of the high concentration resulted in a 23% block of the Ca^2+^ currents (see traces 3, ● and 4, 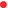). Thus, coupling between both NOP receptors and Ca_V_2.2 channels was maintained as that observed for GRK5 siRNA‐transfected SG neurons. A comparison of the first (●) and fifth (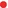) high Noc concentration applications did not result in a significant difference (repeated measures two‐way ANOVA, *p* = 0.07) and is shown in Figure 6A. The summary plot in Figure 6B shows the Noc‐mediated postpulse Ca^2+^ current inhibition or enhancement between the first and fifth application in the siRNA‐transfected SG groups. It can be observed that there was a significantly (*p* < 0.05) lower postpulse Ca^2+^ current inhibition on the fifth Noc application for the neurons transfected with GRK5 siRNA alone or with GRK2 and GRK5 siRNA, while those transfected with scrambled or GRK2 siRNA were indistinguishable. Finally, Figure 6C is a plot of the FR (postpulse/prepulse) measured during the first and fifth exposure to Noc. It can be observed that the mean (±SD) FR was significantly higher (repeated measures two‐way ANOVA) during the first application than the fifth Noc application for the four groups of SG neurons, which suggests that there was an attenuation of the Gβγ‐mediated voltage‐dependent Ca^2+^ channel inhibition following the fifth application.

## 4 Discussion

In SG neurons, the NOP receptor‐mediated inhibition of Ca_V_2.2 channels occurs through a membrane‐delimited and voltage‐dependent pathway, which is mediated by Gβγ protein subunits (Mahmoud et al. 2012). We previously employed an siRNA knockdown screening to identify the natively expressed G protein subunits involved in coupling of NOP receptors to Ca_V_2.2 channels. We observed that Gα_i3_, Gβ_2_, Gβ_4_, and Gγ_7_ were responsible for the inhibition of the Ca^2+^ currents. In the present study, a similar approach was undertaken to identify the GRK isoform responsible for mediating the NOP‐induced desensitization of Ca^2+^ current inhibition. There are seven known GRK isoforms that have been classified into three subfamilies. GRK2 and GRK3 are members of the β‐adrenergic receptor kinase (β‐ARK) subfamily, while the GRK4 subfamily members include GRK4, GRK5, and GRK6 (Penela et al. 2003). GRK2 isoforms are localized primarily in the cytosol, while GRK5 isoforms are membrane‐bound. Our results show that both GRK2 and GRK5 are expressed in SG neurons. Under the “low dose protocol,” a decrease in Ca^2+^ channel modulation from the application of the maximal Noc concentration multiple times was expected to lead to both receptor desensitization and loss of coupling. Under these conditions, desensitization of the response was observed for SG neurons transfected with either scrambled siRNA or GRK2 siRNA. On the other hand, desensitization of GRK5‐ and GRK2&5‐silenced SG neurons was not as pronounced as both groups of neurons maintained their ability to couple NOP receptors and Ca^2+^ channels despite an observable recovery of the prepulse Ca^2+^ currents in the continued presence of an IC_50_ Noc concentration (see time courses Figures 5B and 6A, closed circles). To our knowledge, this is the first report indicating that GRK5 is a physiological regulator of the NOP receptor–Ca_V_2.2 signaling pathway. The GRK5‐mediated desensitization has also been demonstrated for other GPCRs, including muscarinic receptors in the brain (Gainetdinov et al. 1999) and β2‐adrenergic receptors in cardiac muscle (Wang et al. 2008).

As mentioned above, the NOP receptor‐mediated inhibition of Ca^2+^ channels is membrane‐delimited and represents a compact mechanism whereby G proteins bind directly to the channel (i.e., effector). Thus, the proximity of GRK5 within this system suggests this isoform can exert a faster effect when compared to either cytosolic GRK2 or GRK3 isoforms that require a free Gβγ dimer to reach the membrane. Thus, following NOP receptor stimulation, it is more likely that newly released Gβγ will bind to the Ca_V_2.2 rather than interact with GRK2. Furthermore, the readily accessible nature of GRK5 to the receptor and channel suggests that receptor phosphorylation and internalization would be faster. Although GRK5 does not contain Gβγ binding domains, there is a calmodulin binding site at the N‐ and C‐termini. The recent work by Benovic and colleagues suggests that GRK5 phosphorylation of GPCR can be modulated by calmodulin (Komolov et al. 2015, 2021). In this scheme, GRK5 phosphorylates GPCR following activation. In turn, the influx of external Ca^2+^ or the release of internal Ca^2+^ ions from internal stores promotes the binding of calmodulin to GRK5. This leads to GRK5 internalization so that the kinase is unable to phosphorylate GPCRs, though it acquires the ability to phosphorylate cytoplasmic proteins (Komolov et al. 2015, 2021). The identification of these cytoplasmic proteins is unknown. The role of calmodulin in the NOP receptor–Ca_V_2.2 signaling pathway has yet to be determined.

Our finding that knocking down GRK2 did not overtly affect NOP receptor desensitization and coupling with Ca_V_2.2 is unlike other previous studies that have shown that GRK2 and/or GRK3 mediate the Noc‐induced NOP receptor internalization and desensitization. The cell model systems employed in these studies include BE(2)‐C, SH‐SY5Y, HEK293, and mouse brain (Thakker and Standifer 2002; Zhang et al. 2012; Mann et al. 2019). It is possible that the desensitization of NOP receptors occurs in a GRK‐selective manner that is dependent on tissue type (cell line vs. neuron) or expression system (native vs. heterologous) or biased agonism—an observation described by Schulz and colleagues (Mann et al. 2019). A similar finding has been reported for mu opioid receptors expressed in HEK293 cells (Doll et al. 2012). In this report, mu opioid receptors were preferentially phosphorylated by GRK2 and GRK3 when activated by DAMGO, while the GRK5‐mediated phosphorylation occurred when morphine was applied.

Generally, the start of desensitization pathways described for GPCR indicates that they are initiated by GRK‐mediated phosphorylation of serine (Ser) and threonine (Thr) residues within the carboxyl terminal (Ribas et al. 2007). For NOP receptors, it has been demonstrated that Thr^362^, Ser^363^, and Thr^365^ are important for their internalization (Zhang et al. 2012). A subsequent study, however, found that Ser^346^, Ser^351^, Thr^362^, and Ser^363^ were key sites for receptor desensitization and internalization (Mann et al. 2019). The NOP receptor phosphorylation residues under our experimental conditions were not identified. This is one limitation of the present study. A second limitation that needs to be addressed is the scatter of the Western blotting assays, particularly with GRK2 and GRK5 siRNA‐transfected SG tissue, suggesting incomplete silencing. We attempted to minimize this inconsistency by transfecting the SG tissue employing both lipofection and electroporation. Thus, employing both techniques would increase the likelihood of increasing transfection efficiency. Moreover, the Western blots represent data from SG tissue that is made up of both SG neurons and glial cells. Thus, we cannot rule out that glial cells may be the source of the GRK detected.

It was previously reported that a 60 min exposure of HEK293 cells, stably expressing NOP receptors, to 10 μM Noc at 37°C led to the internalization of approximately 50% of receptors, whereas 15% of receptors were internalized after a 10 min exposure (Mann et al. 2019). A study by Meunier and colleagues (Corbani et al. 2004) measured the internalization of stably transfected HEK293 cells with fluorescently tagged NOP receptors at 4°C, 22°C, and 37°C. As expected, half‐maximal NOP receptor internalization was twice as fast (~6 min) at 37°C as that observed at 22 (~12 min) in the continued presence of 0.1 μM Noc. In addition, 100% of NOP receptors were internalized within 20 and 60 min at 37°C and 22°C, respectively (Corbani et al. 2004). A separate study examined Ca^2+^ current desensitization in HEK293 cells acutely transfected with NOP receptor and Ca_V_2.2 cDNA constructs and stably expressing channel auxiliary subunits (Zhang et al. 2012). It was shown that a 30 min pre‐exposure to 1 μM Noc at 37°C led to complete desensitization of NOP receptor‐mediated modulation of Ca^2+^ currents recorded at room temperature. Although the present study did not measure NOP receptor internalization, the Noc concentrations and time scales employed are comparable to those of the aforementioned studies. Thus, it is tempting to speculate that the NOP receptor desensitization observed in our study may have resulted from internalization of the receptors resulting from the continued Noc exposure. For example, Zamponi and colleagues previously found that NOP receptors and Ca_V_2.2 channels formed a complex that internalized following a 30 min exposure to Noc at 37°C (Altier et al. 2006). These observations occurred in both dorsal root ganglion neurons and cells transfected with both proteins. Moreover, it was shown that the Ca_V_2.2 current density was significantly lower in cells exposed to Noc for 30 min, indicative of channel internalization (Altier et al. 2006). In the present study, we did not observe an overt change in Ca_V_2.2 current density following exposure to Noc as the peak Ca^2+^ current returned chiefly to control levels (Figures 3, 4, 5, 6). It is possible that the application of Noc for 30 min may have led to internalization of both NOP receptors and Ca_V_2.2 channels. Alternatively, we cannot rule out involvement of regulators of G protein signaling (RGS) family of proteins, which can accelerate G protein deactivation and play a key role in the Gβγ‐mediated modulation of Ca_V_2.2 channels (Ikeda and Dunlap 1999).

The results obtained with the “low dose protocol” also indicate there was an incomplete prepulse relief of the Ca^2+^ current. These observations suggest that the Noc‐mediated modulation of Ca_V_2.2 channels involves voltage‐dependent and voltage‐independent pathways, which were previously reported to occur with NOP (Beedle et al. 2004) and dopamine D1 receptors (Kisilevsky et al. 2008)—results obtained in heterologous expression systems. Alternatively, the incomplete relief could be explained by the fact that SG neurons express other voltage‐gated Ca^2+^ channels, including Ca_V_2.1 and Ca_V_1.2 (Gilbert et al. 1998; Kukwa et al. 1998). Although the majority of the Ca^2+^ current is carried by Ca_V_2.2, these other channels contribute to the peak Ca^2+^ current and are not modulated by Gβγ as robustly as Ca_V_2.2 channels. It should be noted that, unlike the aforementioned studies, in this set of experiments, the “control” Ca^2+^ current for both prepulse and postpulse currents was acquired in the continued presence of a Noc IC_50_. Under these conditions, the “freely” available Gβγ subunits may not always remain steady, or their affinity for Ca_V_2.2 fluctuates.

In conclusion, this is the first study to demonstrate that GRK5 plays a key role in mediating NOP receptor desensitization of Ca^2+^ current inhibition in SG neurons. Although GRK2 is also expressed in SG neurons, it does not appear to significantly affect this signaling pathway. It remains to be determined whether GRK5‐mediated actions on the NOP receptor and Ca_V_2.2 coupling are modulated by calmodulin. Finally, GRK5 joins a list of other natively expressed proteins we have identified that modulate the coupling of NOP receptors with Ca_V_2.2.

## Author Contributions

**Mohamed Farrag, Marwa Soliman, Saifeldin Mahmoud, Lauren Miller, Paul B. Herold, and Victor Ruiz‐Velasco:** participated in research design. **Mohamed Farrag, Marwa Soliman, Saifeldin Mahmoud, Lauren Miller, Paul B. Herold, and Kristen Brandt:** conducted experiments. **Mohamed Farrag, Marwa Soliman, Saifeldin Mahmoud, Lauren Miller, Paul B. Herold, Kristen Brandt, and Victor Ruiz‐Velasco:** performed data analysis. **Mohamed Farrag, Marwa Soliman, Saifeldin Mahmoud, Paul B. Herold, and Victor Ruiz‐Velasco:** wrote or contributed to the writing of the manuscript.

## Funding

This study was supported by the National Institutes of Health, National Institute on Drug Abuse (Grant DA025574) and National Heart Lung and Blood Institute (Grant HL156513) to V.R.‐V.

## Conflicts of Interest

The authors declare no conflicts of interest.

## Supporting information


**Data S1:** jnr70110‐sup‐0001‐DataS1.docx.

## Data Availability

The data that support the findings of this study are available from the corresponding author upon reasonable request.
